# Perspectives on Rebuilding Health System Governance in Opposition-Controlled Syria: A Qualitative Study

**DOI:** 10.15171/ijhpm.2018.132

**Published:** 2019-01-09

**Authors:** Yazan Douedari, Natasha Howard

**Affiliations:** Department of Global Health and Development, London School of Hygiene and Tropical Medicine, London, UK.

**Keywords:** Health System Governance, Health System Strengthening, Conflict, Resilience, Syria

## Abstract

**Background:** Ongoing conflict and systematic targeting of health facilities and personnel by the Syrian regime in opposition-controlled areas have contributed to health system and governance mechanisms collapse. Health directorates (HDs) were established in opposition-held areas in 2014 by the interim (opposition) Ministry of Health (MoH), to meet emerging needs. As the local health authorities responsible for health system governance in opposition-controlled areas in Syria, they face many challenges. This study explores ongoing health system governance efforts in 5 oppositioncontrolled areas in Syria.

**Methods:** A qualitative study design was selected, using in-depth key informant interviews with 20 participants purposely sampled from HDs, non-governmental organisations (NGOs), donors, and service-users. Data were analysed thematically.

**Results:** Health system governance elements (ie, strategic vision, participation, transparency, responsiveness, equity, effectiveness, accountability, information) were considered important, but not interpreted or addressed equally in opposition-controlled areas. Participants identified HDs as primarily responsible for health system governance in opposition-controlled areas. Main health system governance challenges identified were security (eg, targeting of health facilities and personnel), funding, and capacity. Suggested solutions included supporting HDs, addressing health-worker loss, and improving coordination.

**Conclusion:** Rebuilding health system governance in opposition-controlled areas in Syria is already progressing, despite ongoing conflict. Local health authorities need support to overcome identified challenges and build sustainable health system governance mechanisms

## Background

Syria, a lower-middle-income country in the Eastern Mediterranean, had a population of nearly 21 million in 2010.^1^ The uprising started in 2011, after the arrest and torture of 15 boys in Dara’a who sprayed school walls with anti-government slogans.^1^ Initially non-violent protests soon spread, the Syrian regime responded by arresting and killing protesters,^2-5^ civilians began arming themselves,^6^ and non-violent resistance became armed conflict.^7^ As opposition forces took control of many areas, the regime began bombing people and infrastructure.^8,9^

Authors selected the term ‘opposition-controlled areas’ to refer to areas of Syria controlled militarily by civil and former Syrian army groups (eg, Free Syria Army) in active conflict with the Syrian regime, but not those areas controlled by Kurdish People’s Protection Units (YPG) or foreign forces, eg, ‘Islamic State.’

After conflict onset, the health system in opposition-controlled areas largely collapsed as the 2 most important health governance actors – the state and the World Health Organization (WHO) – no longer operated there.^10^ Health professionals and facilities in opposition-controlled areas appear to have been systematically targeted by the regime and its allies.^11^ As of September 2018, 452 health facilities had been attacked and 886 health-workers killed, at least 90% by Syrian and Russian forces in opposition-controlled areas.^12^ Attacks caused facilities to reduce services or close.^13^ Many health-workers fled, further reducing numbers in opposition-controlled areas. For example, pre-revolution Aleppo had approximately one doctor per 800 people, while opposition-controlled eastern Aleppo had approximately 1 per 7000 in 2015.^13^ However, while deliberate attacks make headlines, the health system has been severely damaged in less obvious but potentially more devastating ways. Sometimes referred to as ‘the weaponisation of health,’ these include government ceasing to fund the health system in opposition-controlled areas and forbidding humanitarian aid or actors from working in these areas, collateral destruction, and mass displacement (including of health-workers).^14^ The loss of state and WHO support and restricted activities of traditional humanitarian actors in opposition-controlled areas contributed to a power vacuum that has been filled by a variety of existing health system and grassroots actors.

The conflict has both ‘weaponised’ and politicised health. Health systems are a natural arena of contestation during civil conflict, both as a means of punishing rebelling population groups (eg, through withdrawal or destruction of services) and as a potential source of legitimacy for opposition actors (eg, if they can provide decent health services they can improve perceived competence, caring, and thus legitimacy). However, health services provision in opposition-controlled areas is challenging, often lacking specialist staff, necessary equipment, and follow-up care.^13,15-17^ Acute and chronic illnesses have increased and many patients die from treatable conditions.^18-20^ Shortages and substandard medical equipment and supplies are worsened by insufficient electricity, water, and generator fuel.^13,18,21^ Professionals often stay 24/7 in health facilities to respond to emergencies, causing high stress and burnout.^13,22,23^ Financial incentives have largely disappeared as salaries are low and irregular.^13^ Limitations are partly overcome by referring some cases to neighbouring countries.^24^ However, this option is not available in all opposition-controlled areas as some are besieged or lack safe international access.^25,26^

These challenges are not unusual, as civilian healthcare provision during active conflict is often constrained. For example, health professionals in Yemen also risk their lives to provide care, within a health system that has almost collapsed, and must focus on emergency provision while managing consequences of health-workers’ migration, shortages, and targeting of facilities and personnel.^27,28^ However, the scale of health system attacks remains greater in Syria, with approximately 485 facility attacks in Syria since 2014 compared to approximately 160 in Yemen since 2015.^12,29,30^ Seeking healthcare across borders was observed in chronic conflicts, including Palestine and Somalia.^31^ No data were found on the emergence of the private sector in opposition-controlled Syria, as has been reported in other conflict-affected countries.^31^

Overall, opposition-controlled areas in Syria are not as disorganised as might be expected.^32^ Existing elements of civil governance include police forces, judiciary structure, and technical directorates, eg, health and education.^33^ These local authorities were mainly formed to meet emerging community management needs, and their quantity, quality, activities, and composition varies.^34^ However, the minimal literature on governance in opposition-controlled areas in Syria does not describe health system governance.^32-36^

Health services were provided by Health Directorates (HDs) and non-govermental organisations (NGOs).^31,37^ International non-governmental organizations and local non-governmental organizations began operating in opposition-controlled areas in late 2012, bringing international funding.^21,38^ Most NGOs provided emergency humanitarian assistance, though some gradually started development and resilience programmes. As no unified formal authority existed in these areas, NGOs often worked without formal oversight or contractual agreements/memorandum of understanding with regime or opposition authorities.^33,39^ HDs in opposition-controlled areas were established in 2014 by the interim Ministry of Health (MoH) affiliated with the National Coalition of Syrian Revolution and Opposition Forces.^40^ Similar structures existed before the conflict, and in regime-controlled areas, but had collapsed in opposition-controlled areas. HDs in opposition-controlled areas aim to replicate the role of their predecessors, in providing provincial health system governance in affiliation with the interim MoH.

HDs are the local authorities responsible for health system governance. However, while HDs were intended to be governance-oriented entities, most have naturally focused on service delivery during the crisis. As essential service providers, HDs face many challenges, including lack of expertise and managerial structures, as most leaders have not held similar positions before, and insufficient financing for required services and salaries.^34^ Daily shelling by the Syrian regime on opposition-held areas has increased instability and personnel loss, making daily life and administration difficult.^34^

Good governance is credited with preventing conflict, reducing poverty, and promoting development. Governance is defined by the UK Department for International Development as “*how institutions, rules and systems of the state-executive, legislature, judiciary, and military operate at a central and local level, and how the state relates to individual citizens, civil society and the private sector.*”^41^ Health system governance can be defined as *“the actions and means adopted by a society to organize itself in the promotion and protection of the health of its population.”*^42,43^ While researchers recently developed this concept further, global research on health systems governance is still relatively limited.^44-49^ However, research indicates that strengthening health system governance in conflict-affected countries can improve resilience and post-conflict rebuilding.^50-52^

For the international community to better support future governance initiatives in Syria, existing governance structures and perspectives on how health system governance is being enacted in opposition-controlled areas require examination. Thus, this study aimed to explore health system governance elements, challenges, and potential solutions in opposition-controlled areas in Syria in 2016, to increase understanding of the contextual and historical factors of local governance engagement in Syria^35^ and contribute to the global debate on health system governance efforts in areas of ongoing conflict. Objectives were to examine local healthcare provider and service-user perceptions of: (*i*) health system governance elements drawn from Siddiqi and colleagues’ framework^43^; (*ii*) roles and relationships of institutional actors, and (*iii*) challenges and potential solutions. Recommendations for policy-makers and practitioners working on health system governance in similar settings are discussed.

## Methods

### Study Design and Sampling

Due to the exploratory nature of research, a qualitative study design was chosen, using semi-structured key informant interviews with health service providers and service-users in selected catchments.^53^ The primary research questions was: “*What lessons can be learnt from health system governance initiatives in opposition-controlled areas in Syria that might be applied to other conflicts*?” The secondary research question was: “*How have local health system actors filled the governance vacuum in opposition-controlled areas of Syria during the conflict, what sorts of health system governance structures exist, and should they be supported*?”

Interview topic guide development and initial analysis were guided by Siddiqi and colleagues’ framework for assessing health system governance in low- and middle-income countries.^43^ Chosen for being relatively straightforward and previously applied in Eastern Mediterranean countries, it includes ten elements: (*i*) strategic vision, (*ii*) participation and consensus orientation, (*iii*) rule of law, (*iv*) transparency, (*v*) responsiveness, (*vi*) equity and inclusiveness, (*vii*) effectiveness and efficiency, (*viii*) accountability, (*ix*) intelligence and information, and (*x*) ethics.^43^ Table 1 in Results section provides a description of each element.

**Table 1:** Health System Governance Elements and Key Findings

**Governance Elements**	**Description**	**Implementation Level Findings**
Strategic vision	• Leaders have a broad long-term perspective on health and human development, a sense of strategic directions for such development, and understanding of historical, cultural and social complexities.	• Long-term vision was limited, but concerned with rebuilding the health system while short-term vision focused on maintaining a semblance of service provision. • Strategic plans ranged from 6 months to 3 years.
Participation and consensus orientation	• All men and women have a voice in decision-making, either directly or through legitimate intermediaries representing their interests.	• Opinions of service-users were sometimes considered, but not routinely. • HDs served as ‘intermediaries’ representing service-user interests.
Rule of law	• Legal frameworks pertaining to health should be fair and enforced impartially, particularly laws on human rights related to health.	• The absence of judiciary, executive, and legislative authorities in opposition-controlled areas severely limited rule of law.
Transparency	• Free flow of information for all health matters, with processes, institutions and information directly accessible and sufficiently informative for those concerned.	• Information was shared internally (eg, within HD departments) and sometimes with external institutional bodies, but little was shared with the public.
Responsiveness	• Institutions and processes should try to serve all stakeholders, to ensure that policies and programs are responsive to service-user needs.	• The health system was perceived by service-users as responsive to people’s needs given the constraints of ongoing conflict.
Equity and Inclusiveness	• All men and women should have opportunities to improve or maintain their health and wellbeing.	• Health services were described positively by service-users as available to everyone for free. • No data were available on potential constraints for marginalised social groups.
Effectiveness and efficiency	• Processes and institutions should produce results that meet population needs and influence health outcomes while making the best use of resources.	• Quality, effectiveness, and efficiency of services were noted by service-users as acceptable given the severe constraints posed by ongoing conflict.
Accountability	• Decision-makers in government, the private sector, and civil society organizations involved in health are accountable to the public and institutional stakeholders.	• Internal and external monitoring and accountability mechanisms existed, eg, ‘beneficiary feedback mechanisms.’ • These were generally noted as insufficient, but improvements were limited by lack of funding and security.
Information and intelligence	• Intelligence and information are essential to provide evidence for informed decisions that support, or do not conflict with, the strategic vision for health.	• Minimal data were provided on types of information collected. • Data collection methods varied, depending on funding, between using specialized teams to collect information directly or depending on other bodies to collect information.
Ethics	• Public health ethics include nonmaleficence, beneficence, dignity, justice, and respect for autonomy, which are important to safeguard service-user interests and rights.	• Ethical considerations were not explored, as this appeared too challenging for participants to address effectively given the ongoing conflict.

Abbreviation: HDs, Health directorates.

Participants were purposively sampled to provide a range of health system contributions, categorised as: (*i*) HD, (*ii*) humanitarian NGO, (*iii*) donor, or (iv) service-user. Recruitment, particularly of HDs, local NGOs and service-users, was challenged by confidentiality and safety concerns, limited electricity/internet access, and time constraints. Personal contacts were thus snowballed using WhatsApp, with each recommending 3 other potential participants, to increase trust and willingness to participate. International NGO and donor participants were recruited through website searches, email invitations, and personal contacts within organisations that had supported health services provision in Syria since at least 2013. NGO and donor participants were sampled nationally, as they normally worked in several provinces. Donors were defined as organisations supporting local authorities, either technically or financially. This approach appeared to provide sufficient diversity given ongoing constraints.

HDs were chosen as local authorities based on expert opinion, with more experienced HD managers (ie, at least 1 year) selected to participate. HD and service-user participants were sampled from 5 opposition-controlled provinces (ie, Aleppo, Dara’a, Hama, Idleb, Rural Damascus). These were chosen, with expert consultation, as the main opposition-controlled areas with tangible forms of governance (Figure). HDs were not homogeneous, with different dates of establishment, sizes, resources, and security levels. For example, some HDs were already 4-5 years old at the time of this study (eg, Idleb), while others were only established the prior year by interim MoH decree (eg, Aleppo). Several HDs had some resources (eg, Idleb), while others had extremely limited funding (eg, Hama). Geographic size and population differed (eg, Idleb HD operated throughout the province, while Hama HD covered less than quarter of the province). Security levels differed in each province, which affected health needs and resources. For example, Rural Damascus HD operated in a besieged area, under constant bombardment and with limited resources, while Dara’a HD covered a relatively stable area and could obtain supplies from Jordan.

**Figure:**
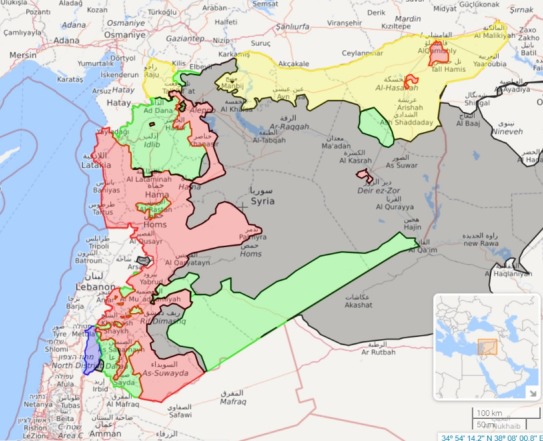
Syria Map Showing Areas of Control in August 1, 2016. Source: https://syria.liveuamap.com/en/time/01.08.2016.

### Data Collection

Two topic guides (ie, providers, service-users) were developed to examine framework elements, governance roles and responsibilities, and perceived challenges/solutions based on the literature and expert consultation, while allowing exploration of emerging concepts.^43^ Interviews were deliberately structured to allow participants to provide their own understanding of governance elements. Thus, *rule-of-law* and* ethics* were excluded after initial question guide testing indicated that they were particularly problematic concepts for participants to consider during conflict. Provider interviews (ie, HD managers, NGO staff, donor representatives) used the full topic guide and lasted approximately one hour. Service-user interviews, using a shortened topic guide (ie, fewer questions per element and excluding *strategic vision* and *intelligence/information*), lasted approximately 30 minutes. As participants were based in Syria or Turkey, and authors in the United Kingdom, all were conducted using Skype, WhatsApp, or telephone.^54^

Twenty interviews were conducted, 5 per participant category, in July-August 2016 (Table 2). Data saturation was determined using a saturation grid as described by Fusch and Ness.^55^ Interviews were conducted at times chosen by participants, digitally recorded and transcribed in Arabic (except one in English) and translated into English. Written and verbal informed consent were recorded before interviews. Participants were sent electronic copies of information sheet and consent form, with questions answered before interviews. Participants without computer access used mobile phones to photograph and send signed consent forms. This worked well as smart phones are readily accessible in Syria. Given security concerns, interviews were recorded anonymously using numerical identification. Recordings and transcripts were stored in password-protected files.

**Table 2:** Participant Information

**Code**	**Category** ^b^	**Location**	**Interview**	**Governance Elements and Themes Discussed**
HD1	Health directorate	Idleb, Syria	WhatsApp	Accountability, strategic vision, transparency, intelligence and information management, legitimacy Roles and relationships of institutional actors Health governance challenges and solutions
HD2	Health directorate	Hama, Syria	WhatsApp	
HD3	Health directorate	Dara’a, Syria	WhatsApp	
HD4	Health directorate	Rural Damascus, Syria	Skype	
HD5	Health directorate	Aleppo, Syria	Skype	
SU1	Service-user	Aleppo, Syria	Skype	Transparency, legitimacy, effectiveness, responsiveness, inclusiveness, participation
SU2	Service-user	Idleb, Syria	WhatsApp­	
SU3	Service-user	Dara’a, Syria	WhatsApp­	
SU4	Service-user	Rural Damascus, Syria	WhatsApp­	
SU5	Service-user	Hama, Syria	WhatsApp­	
NG1^a^	LNGO (active regionally since 2011)	External	Skype	Accountability, strategic vision, transparency, intelligence and information management Roles and relationships of institutional actors Health governance challenges and solutions
NG2^a^	INGO (active regionally since 2013)	External	Skype	
NG3^a^	INGO (active regionally since 2013)	External	Skype	
NG4^a^	INGO (active regionally since 2013)	External	Skype	
NG5^a^	INGO (active regionally since 2012)	External	Skype	
DO1	Donor	External	WhatsApp­	Accountability, strategic vision, transparency, intelligence and information management Roles and relationships of institutional actors Health governance challenges and solutions
DO2	Donor	External	WhatsApp­	
DO3	Donor	External	Skype	
DO4	Donor	External	Skype	
DO5	Donor	External	Phone	

Abbreviations: LNGO, Local Non-Governmental Organization; INGO, International Non-Governmental Organization. ^a^Years in brackets indicate when local and international non-governmental organisations began actively supporting health services provision in Syria; ^b^Interview participants were predominantly Syrian rather than foreign staff, as they were expected to be more familiar with realities in Syria.

### Analysis

Data were analysed thematically by YD using deductive and inductive manual coding, according to the 6 phases described by Braun and Clarke.^56^ Data were initially deductively coded under the ten framework elements^43^ and perceived health system governance: (*i*) responsibilities, (*ii*) roles, (*iii*) challenges, and (*iv*) solutions. Sub-themes were developed inductively as described in Jones et al.^57^ Themes were reviewed by NH and discrepancies agreed between authors. Reporting adheres to COREQ (COnsolidated criteria for REporting Qualitative research).^58^

## Results

Results are organised under health system governance elements and 3 additional themes: (*i*) perceived health system governance responsibility and roles, (*ii*) perceived health system governance challenges, and (*iii*) suggested solutions. Participant responses were integrated where responses were alike and differentiated by participant type when this provided additional nuance.

### Health System Governance Elements

Table 1 provides participant perspectives related to each governance element. Although participants were not specifically asked about rule of law and ethics, relevant data emerging from interviews were included.

Strategic vision was described differently, depending on participants’ role and interpretation, but was by necessity short to medium-term and primarily related to strategic planning. For example, HDs were described as necessarily reactive, trying to maintain immediate service provision despite constraints to longer-term vision due to both physical and funding insecurities. However, some participants described longer-term visions of HDs rebuilding and governing the Syrian health system. HD strategic plans ranged from 6 months to 3 years.

“*We have a strategic plan for 3 years, divided into 3 smaller plans for each year, and we have indicators to measure our achievements at the end of each year that will help us to reach our goal of rebuilding the health system. We want to rebuild the health system according to international standards, taking our war context into account*” (HD5).

NGO participants did not describe an explicit strategic vision, instead discussing intentions to continue service provision and support HDs, though some referred to institutional strategies for regional coordination, developing service provision guidelines, and working with Syrian refugees. NGO strategic plans thus ranged from non-existent to 3 years. Donor participants described their strategic vision as supporting local governance, either as specific HD support or generally supporting the transition from relief to recovery and health system rebuilding, backed by strategic plans ranging from 1 to 3 years.

Accounts differed on the degree of health system participation and consensus, and whether service-user opinions were considered in planning and implementation of services. Most participants indicated that the constraints of working within a conflict-affected area limited opportunities for service-user participation, leaving HDs to act as intermediaries to represent service-user interests.

Given the severe constraints in which facilities operated, participants did not clearly distinguish between responsiveness and effectiveness. All participants indicated that HDs responded to service-users’ needs as much as possible, highlighting that this was not perfect, but good enough considering the situation. Similarly, opinions on effectiveness ranged from “*bad*” to “*excellent.*” However, participants generally agreed that quality was acceptable considering existing challenges.

*“The quality of services is bad. There is an absence of basic [resources] in field hospitals… However, considering the support they [hospitals] receive, I would say it [service quality] is excellent”* (SU3).

Equity and inclusiveness were described minimally but positively, particularly the fact that services were free and available for everyone. However, participants provided no indication of concerns potentially compounded by conflict, eg, among already socially marginalised groups. Participants appeared familiar with transparency issues. HD participants reported that HD governance and service provision data were accessible by all staff, shared with at least one other institutional actor (eg, provincial councils, NGOs, interim MoH, hospital managers), and sometimes with the public. NGOs indicated that they provided public information about service availability, with some providing more comprehensive data, eg, budget, resource allocation, procurement plans, and distribution of beneficiaries. Donors indicated that their websites included data on organisational structure, available and expected funds, decision-making processes, and distribution of funded projects. Some donors additionally shared information with other actors, including HDs, local councils, and vendors. Service-users indicated some level of health system transparency and information access, though details were vague despite probing.

Participants described accountability in terms of internal or external scrutiny and related consequences. HDs were described as having internal and external accountability mechanisms, eg, audits. Some HD participants described electing their HD director as a demonstration of accountability, which they noted as unusual, as before the revolution all staff including directors were appointed. While participants noted existing accountability mechanisms as unsatisfactory, they indicated that significant improvements were not feasible given the lack of security and funding.

“*We have a monitoring and evaluation department in the health directorate [HD] that monitors all projects and ensure that we stick to laws and regulations and international standards […]. We are open for external monitoring and have had external actors monitoring and evaluating our projects in the past, such as one of the NGOs, or donors, or provincial council*” (HD5).

NGO and donor participants described having ‘beneficiary feedback mechanisms’ and internal and external accountability mechanisms, while NGOs additionally mentioned accountability to donors.

Participants described legitimacy as a related concept, in terms of the acceptability and credibility of institutions and their perceived ‘right to govern’ by stakeholders. HDs appeared to gain legitimacy primarily from local actors (eg, health facility managers, local and provincial councils, doctors, communities), though certain external actors (eg, NGOs, donors) could also impart some legitimacy.

“*HD legitimacy exists and continues to exist by agreement of doctors working inside Syria mainly and secondly by working and coordinating with NGOs*” (HD2).

Information management appeared to be a significant issue for all actors. Data were collected, but not necessarily in the most efficient manner, nor could they necessarily be acted upon to support strategy. HDs collected data either by specialist teams or by email from facility managers. NGOs collected data directly from health facilities or from other NGOs, while donors collected data through regular reports from those they funded along with some in-house monitoring and evaluation.

“*We gather information from hospitals by email every month. However, every hospital uses their own form to collect data and this affects the accuracy of [feasibility of consolidating] information gathered*” (HD1).

### Health System Governance Responsibility and Roles

Participants were asked to identify the main institutional actor responsible for health system governance in opposition-controlled areas and describe the roles of relevant actors in health system governance. Most agreed that HDs were responsible for health system governance in opposition-controlled areas, though some HD participants suggested this should shift in future to the interim MoH. However, a few suggested that NGOs were primarily or jointly responsible, since NGOs had the money and provided services. Some NGO participants additionally noted that other local authorities competed for this responsibility in some provinces. Donor participants indicated that several stakeholders were responsible for health system governance, including NGOs, HDs, WHO, and other local actors, but that HDs should be the main responsible actors. Service-user participants said HDs or NGOs were responsible, but suggested HDs should be the responsible body. Responses indicated the fragmented and contested nature of the health system in these provinces as well as pragmatic acceptance that HDs were filling this role as best they could in a challenging environment.

*“There is no one body responsible in Syria now… There is a need for [the interim] MoH and NGOs to share responsibilities for health governance, [as] the concept of governance is new in Syria… I think the current situation is normal, but maybe not healthy […] In the future, the strategic work should be the responsibility of MoH while the NGOs cover the gaps and provide humanitarian work only”* (NG1).

Participants described the interrelated and sometimes contested roles of relevant actors (ie, HDs, interim MoH, NGOs, UN agencies, donors, armed opposition groups) in health system governance. However, discussion was relatively superficial.

Participants highlighted that HDs’ governance role included monitoring other actors’ work, coordinating between actors, representing health professionals, monitoring health programmes, controlling medicines, identifying priorities, health education and granting qualifications, and preparing for post-conflict rebuilding in Syria. Thus, in future, HDs should be responsible to a national MoH, which could merge opposition-controlled and regime-controlled HDs. All NGO participants acknowledged HDs’ roles as local health authorities and NGOs’ partnership relationship, through financial support of HDs, joint programmes, cooperation, and involvement in decision-making. However, several indicated both varied depending how developed each HD was.

“*We coordinate directly and cooperate with HDs, but we do not financially support them because our donors refuse this […]. We depend on HD needs assessment. HD opinion on projects and priorities is very important… The existence of these HDs encourages NGOs to coordinate with each other through the HD*” (NG3).

All donor participants described HDs as the local health authority, though some indicated this role was weak. It was unclear whether this unexpected response was because donor participants informally recognised HDs as local authorities, despite their organisations not providing HDs with the support to act as such, or due to participant bias (eg, assuming this was what they were supposed to say).

Participants agreed that the interim MoH had no current role in health system governance, as it remained weak and its activities had been halted. There was no agreement on whether its role was symbolic or not, due to lack of political and financial support that prevented it fully supporting or being supported by HDs. Similarly, provincial and local councils’ did not have a direct role in health system governance, but were perceived as cooperating and coordinating with HDs, without an explicit executive role. However, some competed with HDs as did some NGOs and donors.

Descriptions of donors were wide-ranging. Some described their governance role as supporting HD governance, eg, by providing salaries, running costs, training, capacity-building, and handing over projects to them. However, few donors supported HDs directly, instead working through NGOs due partially to concerns about treating opposition-controlled health authorities as political substitutes for the Syrian regime. Some donors indicated they should have a direct role in deciding a governance model or discussed political concerns about recognizing HDs as local authorities and consequently a substitute for the Syrian regime. Others indicated donors’ future role should be to provide higher-level support for HDs eg, training on building structures and developing policies or helping HDs to be self-funded. However, most non-donor participants did not describe donors as having a positive role in health system governance, either because donors did not recognise HDs as local health authorities or because donors were more interested in humanitarian response than development. One described donors’ role as negative, each supporting different governance models without coordinating between themselves.

Descriptions of United Nations (UN) agencies’ governance role ranged from none to biased towards the Syrian regime. However, participants indicated that UN agencies could not formally recognise HDs as local health authorities in opposition-controlled areas, as to do so would mean their ejection from regime-controlled areas. However, UN agencies relied on HDs for some projects, eg, polio campaigns, giving HD managers the impression that the UN recognised them as the local health authorities in opposition-controlled areas.

Descriptions of NGOs indicated they had no or a negative role in health system governance, since they provided services regardless of governance. However, NGOs had an indirect role by supporting HDs, either technically (ie, training/capacity-building) or financially, building partnerships to provide health services, or acknowledging HDs as local health authorities in programming. Views were mixed on whether this role would reduce post-conflict. Opinions ranged on the role of armed groups, though most suggested they did not interfere in health system governance. HDs engaged with them as necessary, while NGOs and donors insisted their organisations had no relationship with armed groups.

“*We operate without seeking permission from military factions, because this country is for everyone!*” (HD4)

### Challenges to Health System Governance

Participants were asked about the main challenges to health system governance, with most describing these as security, funding, and HD capacity. Insecurity was related to governance in terms of weakening health system effectiveness, responsiveness, accountability, and intelligence. Security was identified as the major challenge for most participants and the source of other major challenges (eg, loss of health personnel with governance experience). Most discussed the regime’s systemic daily targeting of health facilities and personnel and risks of health-worker kidnappings by armed groups. For example, service-user participants discussed lack of safety in health facilities, due to deliberate targeting by the regime, which resulted in temporary or permanent closure of health facilities, reduced services and capacity, death and migration of health-workers, and destruction of equipment and medicines. One participant mentioned the absence of clinics in his area because of targeting. Another described a hospital being attacked with more than 150 barrel bombs and assassination of a hospital manager by Russian aircraft.

*“They [Syrian regime] are even attacking primary healthcare centres, and even maternity care facilities. These are purely humanitarian facilities and do not treat war-wounded people, and yet they are bombing them”* (HD2).

Unpredictable funding was also related to governance in terms of weakening health system effectiveness, responsiveness, legitimacy, and strategic vision. Challenges were discussed by all except donors. Issues included the unstable funding support for HDs, major discrepancies between HD and NGO staff salaries, and lack of financial support for families of deceased health-workers. NGO participants noted it was easy to access funding for health projects, but very hard to access funding to support health system governance (eg, through providing salaries, training, HD running costs), despite these costs being relatively low, eg, running costs of one hospital were equivalent to the whole provincial HD. Even when health system governance funding existed, it was usually for short periods, eg, 6 months. Additionally, as HDs in opposition-controlled areas were not politically recognised by UN agencies and many donors, receiving and managing funding was difficult.

Capacity was related to governance in terms of weakening health system effectiveness and responsiveness. Most participants discussed the loss of experienced governance professionals and frontline health-workers through death, disability, arrest, or migration that contributed to a lack of governance experience among HD staff. HD participants suggested that this was particularly acute because the prior health system was already weak, lacking effective regulation and distributional equity. As there was no one judicial, executive, or legislative authority, and clarity of HD structures were absent, HDs had to negotiate with all stakeholders, including multiple armed groups, which was difficult and time-consuming.

The effects of reduced capacity on service consistency and quality were major concerns. For example, most HD staff had to work elsewhere to secure income and their limited time reduced the quality of their performance. Similarly, lack of supplies and services was interpreted as reducing quality.

*“Our children have been deprived of polio vaccination for 5 years. This year we were able to get it with the help of NGOs and UNICEF. We still do not have treatment centres for tumours and heart diseases in opposition-controlled areas. We are sending these cases to neighbouring countries”* (HD2).

NGO and donor participants discussed HD weakness and health system fragmentation, including differences between HDs that prevented a unified system, shortages of qualified personnel, and limited overall capacity. While some attributed limited capacity to the targeting and migration of qualified personnel, one suggested that as Syria had been administered centrally, most qualified personnel were in Damascus, under regime control. Participants noted a lack of will to build health system governance, as Syria remained in the emergency response stage and actors – both local and international – were unwilling to work on health system governance building. Others indicated a lack of shared understanding of health system governance among different actors. Descriptions indicated a fragmented and challenging system that was not fully governed by HDs or other actors. For example, participants reported a sometimes complete dependence on NGOs, with each having its own potentially conflicting priorities, and HD competition with local and international bodies that were better established or stronger financially.

*“The whole picture is only seen by HDs. NGOs cannot see the whole picture”* (HD1).

All service-user participants discussed capacity as weakening governance by reducing effectiveness, responsiveness, and legitimacy (eg, through insufficient supplies and health-workers – especially doctors). It was difficult to find medicines, which even when available were not affordable, and chronic care suffered. One described visiting a doctor for consultation, who was seeing 3-4 patients at the same time and giving painkillers for non-emergency cases. Thus, capacity at health facilities was limited.

*“My hand was injured and fractured by shelling shrapnel […] I had to give first aid to myself…. When I went to the hospital they gave me painkillers, cooled down my situation, and sent me home […] there were hundreds of injured people…They were too busy with other injuries…”* (SU4).

### Potential Solutions

Participants suggested 3 main approaches to strengthening provincial health systems governance: (*i*) supporting HDs, (*ii*) mitigating health-worker losses, and (*iii*) coordination.

#### Supporting Health Directorates

All participants agreed financial support was needed for HDs to provide meaningful governance in opposition-controlled areas. Existing largely ad-hoc funding mechanisms included collecting fees for services such as licensing or medicine testing, reintroducing symbolic user fees, developing income-generating projects, finding staff part-time jobs (eg, with NGOs), volunteering their time with HDs where possible, and shifting funds from other programmes. For example, financing for deceased health-workers’ families involved diverting a percentage of staff salaries to a support fund. No methods were completely successful. HDs took advice from experts living outside Syria and depended on WHO and the United Nations International Children’s Emergency Fund (UNICEF) regulations on strengthening health systems.

NGO and donor participants discussed means of supporting HD governance, including financial support for staff salaries and running costs, technical support through training and capacity-building, and symbolic support by working with HDs as the recognised local health authorities. Symbolic support included increasing the visibility of HDs, using HD statistics, allowing HDs to monitor NGOs, representing HDs in the health cluster, involving HDs in decision-making, marketing HDs to donors, and handing over some tasks. Some NGOs had signed an agreement to support HDs and recognise them as the only local health authority in opposition-held areas.

“*In our exit plans, we always ensure that health projects should be handed over to HDs*” (DO3).

#### Mitigating Health-Worker Losses

HD and donor participants identified mitigation of losses as crucial to governance, while NGO and service-user participants did not mention this. One participant described an 8-month health education initiative, to train new health-workers to fill gaps. Called “training while working,” it was initiated to train and certify as ‘Nurse Assistants’ those working without qualifications in the health sector. It did not cover the medical gap (medical students need 6 years training and four years’ specialisation), but could encourage other health professionals to stay in the country since they could continue studying and progressing. Another participant addressed health-worker emigration and loss by increasing the workload of existing doctors, with each working in several hospitals during the week. This increased burnout, worsening health system effectiveness and responsiveness. Another reported seeking advice from colleagues in other HDs to compensate for his own lack of leadership and governance experience. Donor participants discussed minimising expertise migration through financial incentives. Suggestions included providing employment or salaries where none existed, providing higher salaries for doctors in certain areas, and providing medical training to compensate for losses.

#### Coordination

Participants suggested that coordination with other actors could help address health system fragmentation and competition in the absence of a united authority. NGO and donor participants stressed the importance of coordination among health actors, especially HDs, NGOs, and local councils.

“*We insist on coordinating with HDs in all matters even if we could solve issues more quickly through our partners without the HDs*” (NG3).

Participants mentioned coordination through the humanitarian health cluster in Gaziantep Turkey, but highlighted that it gave HDs only one seat rather than a seat for each HD, which was described as insufficient given their differing needs and contexts.

## Discussion

### Key Findings

This study adds to research on governance in opposition-controlled areas in Syria and provides insights into health system governance experiences during conflict. Similar research on Syria focused on general governance or health needs, making this the first study authors are aware of examining health system governance in opposition-controlled areas and including a range of health system voices (ie, local health authorities, NGOs, donors, service-users). This may be the first study exploring Syrian service-user perspectives on health or governance since conflict began and is an initial attempt to explore health system governance during conflict, as previous research focused on ‘post-conflict’ reconstruction without considering efforts begun during conflict.

All provider categories (ie, HDs, NGOs, donors) reported some level of strategic vision, accountability, transparency, and information management. Strategic vision focused on building local governance and continuing services provision. Short-term focus in strategic vision and planning is consistent with the High-Level Forum model and other literature on effects of chronic conflict on health system components.^59,60^ Agreement among HDs and service-users that legitimacy was gained from local actors is consistent with governance research in Syria.^35^ Service-user agreement on health sector responsiveness and transparency, and varied opinions on effectiveness and participation, is consistent with Khalaf’s argument that perceived effectiveness in Syria is based on service delivery.^35^ Thus, using Siddiqi and colleagues’ health system governance elements as qualitative measures appeared understandable to participants and was consistent with the literature.^43,47,61-63^

Emergence of grassroots governance was also observed during chronic conflicts, such as in Palestine and Somalia.^31^ Importantly, HDs were most frequently identified as the bodies responsible for health system governance among all participants, with NGO and donor participants describing their role as supporting HDs in health system governance, which was not found in the literature. The interim MoH was described as having no meaningful role in health system governance, possibly due to its inability to support HDs financially, or reflecting the complex relationships with other actors. While observers often describe the emergence of HDs as a grassroots phenomenon, independent from interim MoH decisions, the reality is more complex. For example, though HDs were officially established by the interim MoH, they did not receive financial or technical support from it and therefore some HDs grew independently. Others existed before the interim MoH decree (eg, Idleb HD).

NGOs potentially negative role was consistent with the literature.^64-68^ For example, Haar argued that NGOs not coordinating project planning and implementation with local authorities negatively affected local authorities’ legitimacy and stability.^66^ Donor roles in supporting health system governance and coordination in fragile states has been highlighted,^69^ making it interesting that NGO and donor participants both suggested the other had no positive role in health system governance in Syria. Significant disagreement about UN agency roles was apparent, indicating tensions and contested perspectives within the international humanitarian community. Participant accusations of UN bias towards the Syrian regime was consistent with recent research.^14^ While the literature supported findings of interference by armed groups, most participants said armed opposition groups generally did not interfere in health system governance.^13^

Health system governance challenges identified (ie, security, funding, capacity) were consistent with the literature, which also discussed targeting of health facilities and personnel, health-worker losses, lack of funding, and contestation between health system governance actors.^13,34,66^ Other “weaponisation of health” challenges include the Syrian regime halting health system financing in opposition-held areas and preventing humanitarian actors from working there.^14^ Differences in the perceived importance of challenges between participant categories are worth noting. For example, while security appeared crucial for HD and service-user participants, it was less discussed by NGO and donor participants. A potential reason was that HDs and service-users resided in Syria, while NGOs and donors were generally based outside the country. Funding seemed crucial for HD participants and less so for NGO and donor participants, perhaps because the latter had greater funding access. All mentioned capacity as a governance challenge, indicating its overall significance. Interestingly, HD participants highlighted lack of support, while NGOs highlighted lack of will to support HDs and build health system governance, and donors highlighted HD competence. These differences indicated a vicious circle in which HDs needed support to overcome weaknesses and build capacity, while donors were unwilling to support HDs because they lacked capacity. Haar also suggested that donors perceive investment in fragile states as politically and financially risky, as such countries have relatively poor policies and institutions.^66^

The main solutions discussed by participants (ie, supporting HDs, mitigating health-worker losses, coordination) were consistent with existing literature.^13,34,35,66,69,70^ For example, Baker discussed establishing a nursing school in Aleppo.^13^ Aljundi discussed the need for financial and technical support for local authorities in opposition-controlled areas.^34^ Khalaf argued that economic and human resources were essential for governance success.^35^ It is worth noting that, though participants focused on how HDs could be supported, HDs were not merely passive recipients of interventions and could potentially do more themselves to engender support. However, it was also true that HDs had a very constrained policy space for manoeuvre. Thus, it is possible that provision of appropriate external support could potentially do more to improve their circumstances than changes within HDs themselves.

Since research was conducted, health system governance in Idleb and Hama remains relatively unchanged. Two of the study provinces (ie, Rural Damascus, Dara’a) were retaken by Russian and Syrian regime forces in 2018. All their local authority structures, including HDs, were dismantled for ‘defying’ Syrian regime authority. In late 2016, Aleppo city and areas of surrounding countryside was retaken by Russian and Syrian forces, with similar treatment of HDs. In 2017-2018, Turkey took control of northern Aleppo (ie, Euphrates Shield) and northwest Aleppo (ie, Kurdish-populated Afrin district), which had been controlled by Islamic State (IS) and by Kurdish YPG respectively. Health system governance in these 2 areas is now being led by the Turkish MoH.

### Implications for Policy and Practice

Findings suggest several potential implications for policy-makers (eg, recognising and supporting HDs’ health system governance role, considering long-term needs when allocating funding, advocating against health facility targeting), donors and practitioners (eg, supporting HD capacity-building and running costs, supporting health-worker education and training, improving coordination), and researchers (eg, identifying and examining health system governance initiatives in conflict-affected and opposition-controlled areas that can be built upon as violence reduces or ceases).

Given the agreed importance of health system governance globally and the reality that it receives little funding or consideration in Syria, decision-makers could acknowledge and better support HD health system governance efforts. This would enable longer-term funding perspectives and greater strategic vision. However, to do this, the international community needs to accept HDs as the de facto health authority in opposition-controlled areas.

Destruction of health facilities and personnel was considered a major challenge and a potential war crime. Humanitarian and security decision-makers globally must act more strongly to protect health facilities and personnel during conflict.

Donors could strengthen existing health system governance by strengthening HDs, eg, building their capacity to be financially independent, and in the interim supporting HD salaries, running costs, and/or channelling health projects through them until they are strong enough to be independent. Ideally, some emergency response funding could support health system governance. Education initiatives need political, financial, and technical support to succeed. An example of a successful initiative that could be adapted is community midwifery education, eg, in Afghanistan.^71,72^ All practitioners, including HDs, should coordinate more deliberately at local levels, to overcome the challenges of fragmentation and multiple competing actors and interests to rebuild a unified health system.

Further research is needed to examine health system governance elements, development, and initiatives in opposition-controlled areas and the perspective of a broader range of actors, particularly UN agencies. Research could compare other countries with ongoing conflict (eg, Somalia, South Sudan) to help generalise lessons learnt. Additionally, given the rapid changes in Syria, further qualitative research on the evolving situation will be needed. Study findings show that health system governance may begin while a conflict is ongoing, indicating that a broader research agenda on health governance emergence, development, effective elements and structures during conflict would be valuable.

### Limitations and Strengths

This study had several limitations. First, health system governance is a complex concept for which data in Syria were extremely limited. Siddiqi and colleagues’ framework provided a useful starting point, but was insufficiently flexible for un-adapted use in conflict-affected settings. Further work would be needed to adapt it for use in these settings. As participants were encouraged to provide their own interpretations of key terms, to facilitate relaxation and rapport, some limitations existed in translations as some had no precise equivalent in Arabic. For example, ‘accountability’ in Arabic gives a sense of investigation and lack of trust (the term was not used in pre-revolution Syria), while ‘legitimacy’ has religious connotations but can be understood as in English. Thus, though participants were familiar with governance concepts and terms, some responses were limited or superficial due to differential understanding or lack of in-depth knowledge. Similarly, excluding questions on some governance elements (eg, ethics) limited what data could be included in Table 1 on these concepts.

Second, time and funding constraints prevented inputs from additional health system governance actors (eg, UN agencies, local/provincial councils, interim MoH, opposition armed groups) or exploration of inter-provincial differences between HDs. Similarly, detailing and distinguishing between the roles and capacities of local and international NGOs was beyond the scope of this research.

Third, conducting interviews remotely could have influenced responses, eg, in terms of rapport, non-verbal cues, and additional ethics considerations of using third-party software. However, similar to findings by Lo-Iacono et al, authors found that using WhatsApp or Skype and additional ethics requirements did not appear to noticeably reduce participant candour or observed rapport.^54^ Finally, the situation in Syria is fluid, though results could still be relevant for areas of ongoing conflict and non-state control.

This study had several methodological strengths. First, the lead author was a Syrian health-worker who provided frontline healthcare in opposition-controlled areas during the current conflict and thus had solid insider knowledge of contextual and cultural issues. Second, HD participants and service-users were chosen from 5 opposition-controlled provinces to increase representation and explore issues in all opposition-controlled provinces. Third, major institutional actors in health system governance in opposition-controlled areas of Syria participated. Finally, inclusion of service-users in opposition-controlled areas was particularly important as service-user voices can be a particularly challenging to access during conflicts.

### Final Thoughts

A purported reason for the Syrian uprising was as a popular revolution to create a more equitable country. Despite almost seven years of fighting, participants were clear that hope remains. Health system governance rebuilding has already progressed in opposition-controlled areas. Waiting until conflict ends to consider health system governance could irreparably weaken these fragile initiatives. Local HDs need specific political, technical, and financial support if they are to overcome ongoing challenges and develop sustainable governance.

## Acknowledgements

Thanks to study participants, who provide health services in areas of considerable physical risk.

## Ethical issues

Approval was provided by the MSc Research Ethics Committee of the London School of Hygiene & Tropical Medicine in the United Kingdom. As no formal ethics committee or internationally-recognised government institutions operated in opposition-controlled areas in Syria at the time of research, local approval was obtained from HDs. Written and verbal informed consent was recorded from all participants before interview.

## Availability of data

The dataset generated and analysed during this study are not publicly available due to participant confidentiality, but transcript portions can be made available from the corresponding author on reasonable request.

## Competing interests

Authors declare that they have no competing interests.

## Funding

Chevening Scholarships, the UK government’s global scholarship programme, funded by the Foreign and Commonwealth Office (FCO) and partner organizations provided some study funding as part of the lead authors’ MSc studies. The funder had no role in study design, data collection, analysis, interpretation, or manuscript writing.

## Authors’ contributions

YD designed the study, collected and analysed data, and drafted the manuscript. NH contributed to study design and data interpretation and critically revised the manuscript. Both authors approved the version for submission.

## Key messages

**Implications for policy makers**

- Political recognition of local health authorities is necessary if initial grassroots governance initiatives are to succeed.
- Financial and technical support of local health authorities is urgently needed if they are to survive.
- To protect fragile health system governance initiatives, the international community must do more to end bombings of health facilities and health-workers in opposition-controlled areas.

**Implications for public**

This qualitative study is a first effort to explore health system governance in opposition-controlled areas in Syria. found that despite tremendous challenges, including ongoing conflict and targeted bombings, local health directorates (HDs) in opposition-controlled areas are continuing to provide health services and developing fragile health system governance initiatives. Rebuilding of health system governance has already started, through local initiatives in opposition-controlled areas in Syria, but these HDs require additional technical and financial support to ensure that progress is not lost.
